# Analysis of controlled ovarian hyperstimulation protocols in women over 35 years old with poor ovarian response: a real-world study

**DOI:** 10.1186/s12884-023-06112-4

**Published:** 2023-11-23

**Authors:** Xiaoyan Duan, Zhan Li, Mingming Li, Xing Ma

**Affiliations:** 1 Department of Gynaecology and obstetrics, The First People’s Hospital of Shangqiu, 292 Kaixuan South Road, Henan, Shangqiu People’s Republic of China; 2 Graduate School of Zhengzhou University, Henan, People’s Republic of China

**Keywords:** Poor ovarian response, Controlled ovarian hyperstimulation, Age, Assisted reproductive technology, Real-world study

## Abstract

The objective of this study was to investigate the optimal controlled ovarian hyperstimulation (COH) protocol for patients aged 35 and above with poor ovarian response (POR), utilizing real-world data. This retrospective cohort study examined clinical information from a total of 4256 patients between January 2017 and November 2022. The patients were categorized into three groups: modified GnRH agonist protocol (2116 patients), GnRH antagonist protocol (1628 patients), and Mild stimulation protocol (512 patients). Comparative analysis was conducted on clinical variables and pregnancy outcomes across the three groups. The GnRH agonist protocol was associated with a higher number of oocyte number (4.02 ± 2.25 vs. 3.15 ± 1.52 vs. 2.40 ± 1.26, *p* < 0.001), higher number of transferable embryos (1.73 ± 1.02 vs. 1.35 ± 1.22 vs. 1.10 ± 0.86, *p* = 0.016), higher cumulative live birth rate 28.50(603/2116) vs. 24.94(406/1628) vs. 20.51(105/512), *p* < 0.001) than GnRH antagonist protocol and Mild stimulation protocol, the Mild stimulation protocol was associated with a higher miscarriage rates 16.27(62/381) vs. 16.61(48/289) vs. 32.22(29/90), *p* = 0.001) than the other two groups. Therefore, it can be concluded that all three protocols can be used in patients over 35 years old with poor ovarian response. However, if patients require more frozen-thawed embryo transfers to achieve better cumulative live birth rates, the modified GnRH agonist protocol may be the preferable option.

## Introduction

The development of an effective protocol for patients aged 35 and above with poor ovarian response (POR) who are undergoing assisted reproductive technology (ART) remains a significant challenge [1, 2], These patients experience a decline in ovarian reserve, which hinders their ability to achieve successful pregnancy outcomes. Tailored controlled ovarian hyperstimulation (COH) protocols are crucial for improving pregnancy outcomes in this population [3, 4]. Multiple strategies exist for managing ovarian hyperstimulation, encompassing diverse gonadotropin preparations, dosages, durations, and the incorporation of adjuvant therapies. However, the effective protocol remains elusive for older patients with POR [5]. The utilization of controlled ovarian hyperstimulation protocols is of utmost importance in ART and has a profound impact on the success rate of in vitro fertilization (IVF) and intracytoplasmic sperm injection (ICSI) treatments. Determining the most suitable ovarian stimulation protocol for women of advanced age with POR involves considering various factors, including age, ovarian reserve, and previous response to ovarian stimulation [6–8]. The GnRH agonist protocol, GnRH antagonist protocol, and Mild stimulation protocol have been extensively examined in both international and domestic studies for patients aged 35 and above with POR. However, there exist significant controversies regarding their effectiveness, safety, and the resulting pregnancy outcomes [9, 10].

The GnRH agonist protocol has gained significant global usage in IVF cycles due to its efficacy in regulating follicular growth and promoting a predictable and synchronized ovarian stimulation cycle [11–13]. Nevertheless, ongoing discussions persist regarding the effectiveness and safety of this protocol [14]. GnRH antagonist protocols aim to inhibit the secretion of gonadotropins from the pituitary gland to prevent premature ovulation. In comparison to the agonist protocol, the rapid onset and shorter half-lives of GnRH antagonists lead to decreased levels of E2, potentially mitigating the likelihood of ovarian hyperstimulation syndrome [15, 16]. However, certain researchers have documented decreased rates of successful live births associated with the antagonist protocol due to untimely regression of the corpus luteum [17]. In an effort to mitigate the adverse effects of a high-dose gonadotropin regimen, such as ovarian hyperstimulation syndrome (OHSS), premature luteinization, and suboptimal oocyte quality, Mild stimulation protocols have been devised, nevertheless, debates persist regarding the efficacy of Mild stimulation protocols in enhancing the outcomes of assisted reproductive technology [18, 19]. It is therefore necessary to conduct further studies on these topics.

Considering the drawbacks associated with controlled ovarian hyperstimulation protocols for individuals aged 35 and above, such as low egg collection and pregnancy rates, as well as a high abortion rate, we developed an enhanced regimen. In a preliminary experiment conducted at our center, we implemented a modified GnRH agonist protocol specifically for patients over 35 years old with poor ovarian response. This protocol, referred to as the early-follicular-phase long-acting GnRH-a long protocol, yielded satisfactory pregnancy outcomes and has since been widely adopted as the predominant approach in numerous reproductive medicine centers in China, due to its enhancement of endometrial receptivity, clinical pregnancy rates and its reduction of the abortion rate in the normal patient population. However, the data collected did not meet the requirements for statistical analysis, prompting the need for the current study. The objective of this study is to examine the latest evidence-based guidelines for customized COH in patients with POR, as well as to investigate various stimulation protocols and their effects on pregnancy outcomes. Additionally, this study aims to offer valuable insights for clinicians in their treatment strategies for these patients.

## Materials and methods

This real-world study was conducted using data from the First People’s Hospital of Shangqiu, spanning from 1 to 2017 to 30 November 2022. A total of 4256 Patients over 35 years old with poor ovarian response were included in this study. The experimental materials utilized in this study were obtained from the Electronic Medical Record Cohort Database of the Reproductive Medical Center of the First People’s Hospital of Shangqiu, inclusion criteria were adult women aged 35 years and above and diagnosed with poor ovarian response according to the Poseidon criteria. Patients were followed up until September 2023. Two independent investigators retrieved and reviewed the medical records of all eligible patients. Relevant information such as demographic details, clinical and laboratory characteristics, and treatment outcomes were extracted. This study was approved by the Ethics Committee of the First People’s Hospital of Shangqiu (SQ20190016). This study followed a retrospective cohort design, and the need for informed consent was waived by the Medical Ethics Committee of the First People’s Hospital of Shangqiu.

Eligible subjects are patients with low ovarian response as defined by the POSEIDON criteria, including: POSEIDON group 2: age ≥ 35 years, AFC ≥ 5, AMH ≥ 1.2 ng/mL and ≤ 9 oocytes retrieved in the first stimulation cycles and POSEIDON group 4: age ≥ 35 years, AFC < 5, AMH < 1.2 ng/mL. For the purpose of our study, we have used the following criteria to define Ovarian Hyper-stimulation Syndrome (OHSS), as long as any of the following diagnoses are met, we assume that the patient has OHSS. (1) ovary enlargement, bloating, mild abdominal pain, and ovary diameter less than 8 cm; (2) severe bloating, nausea and vomiting, presence of ascites, and ovarian diameter ranging from 8 to 12 cm; 3). Tension ascites, HCT > 0.55, WBC > 15*10^9^/L, oliguria / anuria, vascular embolism, acute respiratory distress syndrome.

Modified GnRH agonist protocol: The protocol involved the administration of 3.75 mg of GnRH-a (Diphereline, Beaufort-Ipson, France) on days 2–4 of menstruation. Additionally, the patients underwent serum sex hormone level measurement and ultrasound monitoring. Ovarian stimulation was initiated when FSH < 5 IU/L, LH < 5 IU/L, estradiol < 30 g/mL, and progesterone < 1 ng/mL were observed, along with follicle sizes of 3–5 mm by ultrasound. This was achieved by administering recombinant follicle-stimulating hormone (rFSH; Gonal F, Merck Serono, Switzerland) at a starting dose of 125–300 IU/day on day 2 or 3 of the menstrual cycle. An adjustment was made to the FSH dose according to the patient’s age, weight, ovarian reserve, and previous response. Follicular development was monitored through daily transvaginal ultrasonography and serum hormone measurements, with adjustments to the FSH dose as necessary. Once one or more follicles reached an average diameter of 18 mm, ovulation was induced by administering 5000-10,000 IU of human chorionic gonadotropin (hCG; Livzon, China). Following the ovulation trigger, luteal phase support was provided by administering oral dydrogesterone (Duphaston; Abbott, USA) or progesterone (XianJu Pharma, China) starting from the day after ovulation trigger until a negative pregnancy test or up to 12 weeks of gestation if pregnancy was confirmed.

GnRH antagonist protocol: patients were subcutaneously administered with follicle-stimulating hormone (rFSH; Gonal F, Merck Serono, Switzerland) for a duration of 5–7 days. The initial dosage of FSH was determined by the clinician, taking into consideration the patient’s age, body mass index, and antral follicle count. Subsequent adjustments to the dosage were made based on the follicular response, which was monitored through transvaginal ultrasound. The introduction of the gonadotropin-releasing hormone antagonist (Orgalutran, Netherlands) occurred when the leading follicle reached a diameter ranging from 12 to 14 mm. Cetrorelix or ganirelix was administered via subcutaneous injection on a daily basis until the day of hCG (Livzon, China) trigger. Follicular development was monitored using transvaginal ultrasound. Ovulation was induced by administering hCG once one or more follicles reached an average diameter of 18 mm. The dosage of hCG was determined by the clinician, taking into consideration the patient’s age, body mass index, and follicular response. The luteal support scheme employed was identical to the modified GnRH agonist protocol.

Mild stimulation: The patients underwent ovarian stimulation using a Mild stimulation protocol. Commencing on the second day of the menstrual cycle, the patients were subcutaneously administered a low dose of follicle-stimulating hormone (rFSH; Gonal F, Merck Serono, Switzerland) for a duration of 5–7 days. The initial dose of FSH ranged from 75 to 225 IU/day and was adjusted based on the patient’s age, body mass index, and antral follicle count. The progression of follicle development is meticulously observed through the utilization of ultrasound examinations and hormone assessments, specifically monitoring luteinizing hormone (LH) peaks. In the event that an LH peak surpasses 10 IU/L or exceeds twice the baseline value, alongside a follicle size exceeding 18 mm, hCG was administered to trigger ovulation. Conversely, if the follicle size is smaller, the implementation of antagonist (Orgalutran, Netherlands) is employed as a preventive measure against premature ovulation, prompting a transition to an antagonist protocol within our established procedure. The luteal support scheme employed was identical to the modified GnRH agonist protocol.

### Statistical analysis

The statistical software used for all statistical analyses was the Statistical Package for the Social Sciences version 26.0 (IBM Corp., Armonk, NY, USA). Continuous variables were presented as mean ± standard deviation and compared using one-way analysis of variance followed by the Bonferroni post-hoc test. Categorical variables were expressed as frequencies and percentages for each ovarian hyperstimulation protocol and compared using the chi-square test or Fisher’s exact test, as appropriate. The threshold for statistical significance was set at *p* < 0.05.

## Results

This study encompassed a cohort of 4256 patients aged 35 years and older who exhibited poor ovarian response to ART. Among these cycles, a total of 2116 patients underwent ovarian stimulation using the GnRH agonist protocol, GnRH antagonist protocol was carried out in 1628 patient. Mild stimulation protocol was carried out in 512 patients. There were no statistically significant differences observed in baseline characteristics, including age, body mass index, basal follicle-stimulating hormone, basal luteinizing hormone, basal estradiol, anti-Müllerian hormone, thyroid stimulating hormone, serum free triiodothyronine, and serum free thyroxine levels, among patients who underwent the three different protocols (Table 1).

**Table 1:** Comparison of baseline parameters among the three protocols

Protocols	GnRH agonist (n = 2116)	GnRH antagonist (n = 1628)	Mild stimulation (n = 512)	*P* value
Age (years)	39.25 ± 3.05	38.98 ± 2.79	39.71 ± 3.62	0.581
Infertility years (years)	3.61 ± 2.46	3.46 ± 2.52	3.41 ± 2.23	0.285
**Sterility type (%)**				
Primary infertility	60.1(1272/2116)	59.8(973/1628)	58.0(297/512)	0.683
Secondary infertility	39.9(844/2116)	40.2(655/1628)	42.0(215/512)	
BMI (kg/m2)	25.21 ± 2.94	25.25 ± 3.52	25.81 ± 2.95	0.456
Basal FSH (IU/L)	9.66 ± 4.92	10.76 ± 5.32	10.25 ± 5.71	0.253
Basal LH (IU/L)	4.52 ± 2.24	4.84 ± 2.31	4.41 ± 2.25	0.352
Basal E2 (ng/L)	46.81 ± 32.04	44.95 ± 36.5	43.49 ± 30.75	0.795
Basal P (µg/L)	0.57 ± 0.51	0.49 ± 0.31	0.58 ± 0.42	0.305
AMH (ng/mL)	0.67 ± 0.21	0.75 ± 0.31	0.61 ± 0.31	0.152
TSH (mlU/ml)	2.33 ± 1.14	2.42 ± 1.25	2.61 ± 1.54	0.621
FT3 (pmol/L)	5.41 ± 0.91	5.41 ± 0.65	5.56 ± 0.67	0.964
FT4 (pmol/L)	11.34 ± 2.71	12.01 ± 1.99	11.56 ± 2.05	0.520
**Method of fertilization**				
IVF	76.9(1627/2116)	73.9(1203/1628)	76.4(391/512)	0.098
ICSI	23.1(489/2116)	26.1(425/1628)	23.6(121/512)	

BMI, body mass index; FSH, follicular-stimulating hormone; LH, luteinizing hormone; E2, estradiol; P, progesterone; AMH, anti-Müllerian hormone;TSH, thyroid stimulating hormone; FT3, serum free triiodothyronine; FT4, Serum free thyroxine

#*P* < 0.05, vs. GnRH agonist; *P < 0.05, vs. GnRH antagonist

After that, we compared the outcomes of COH in each group based on the number of oocytes and embryos produced. the GnRH agonist protocol was associated with a higher total dosage of Gn used (3952.59 ± 385.13 vs. 3251.85 ± 317.89 [GnRH antagonist] vs. 2696.25 ± 291.81 [Mild stimulation], *p* < 0.001), longer duration of gonadotropin use (14.04 ± 3.02 vs. 11.25 ± 2.96 [GnRH antagonist ] vs. 12.53 ± 2.74 [Mild stimulation], *p* < 0.001), higher number of oocyte number (4.02 ± 2.25 vs. 3.15 ± 1.52 [GnRH antagonist ] vs. 2.40 ± 1.26 [Mild stimulation], *p* < 0.001), higher number of MII number (3.25 ± 2.59 vs. 2.60 ± 1.84 [GnRH antagonist ] vs. 1.93 ± 1.03 [ Mild stimulation], *p* < 0.001), higher number of transferable embryos (1.73 ± 1.02 vs. 1.35 ± 1.22 vs. 1.10 ± 0.86, *p* = 0.016), higher number of good-quality embryos (1.51 ± 0.97 vs. 1.04 ± 0.89 vs. 0.97 ± 0.68, *p* = 0.042), higher OHSS rate (4.63(98/2116) vs. 3.99(65/1628) vs. 2.15(11/512), *p* = 0.038) than GnRH antagonist protocol and Mild stimulation protocol. There were no differences in the oocyte maturation rates, fresh cycle cancellation rate and fertilization rates among patients who underwent the three ovarian hyperstimulation protocols (Table 2).

**Table 2:** Comparison of the outcome of COH in terms of oocytes and embryos among the three protocols

Protocols	GnRH agonist (n = 2116)	GnRH antagonist (n = 1628)	Mild stimulation (n = 512)	*P* value
Starting dosage of Gn used (IU)	262.41 ± 73.55	223.85 ± 62.52#	115.83 ± 58.29#*	< 0.001
Total dosage of Gn used (IU)	3952.59 ± 385.13	3251.85 ± 317.89#	2696.25 ± 291.81#*	< 0.001
Duration of Gn used (days)	14.04 ± 3.02	11.25 ± 2.96#	12.53 ± 2.74#	0.143
Oocyte number	4.02 ± 2.25	3.15 ± 1.52#	2.40 ± 1.26#*	< 0.001
MII number	3.25 ± 2.59	2.60 ± 1.84#	1.93 ± 1.03#*	< 0.001
Oocyte maturation rates (%)	80.84 ± 14.36	81.51 ± 19.85	80.46 ± 15.93	0.931
Transferable embryos	1.73 ± 1.02	1.35 ± 1.22#	1.10 ± 0.86#	0.016
Good-quality embryos	1.51 ± 0.97	1.04 ± 0.89#	0.97 ± 0.68#	0.042
Fertilization rates (%)	63.41 ± 32.52	59.43 ± 31.53	60.62 ± 36.11	0.825
OHSS rates	4.63(98/2116)	3.99(65/1628)	2.15(11/512)#	0.038

MII, metaphase II; OHSS, ovarian hyperstimulate syndrome; #*P* < 0.05, vs. GnRH agonist

*P < 0.05, vs. GnRH antagonist

We then compared the pregnancy outcome of the three protocols in each group, the GnRH agonist protocol was associated with a higher cumulative live birth rate 28.50(603/2116) vs. 24.94(406/1628) vs. 20.51(105/512), *p* < 0.001) than GnRH antagonist protocol and Mild stimulation protocol, the Mild stimulation protocol was associated with a higher miscarriage rates 16.27(62/381) vs. 16.61(48/289) vs. 32.22(29/90), *p* = 0.001) than the other two groups, There were no differences in implantation rates, pregnancy rates per transfer and live birth rates per transfer among the three ovarian hyperstimulation protocols (Table 3).

**Table 3:** Comparison of the pregnancy outcome among the three protocols

Protocols	GnRH agonist (n = 2116)	GnRH antagonist (n = 1628)	Mild stimulation (n = 512)	*P* value
Implantation rates (%)	19.83(596/3006)	20.67(446/2158)	19.53(134/686)	0.702
Pregnancy rates per transfer (%)	18.01(381/2116)	17.75(289/1628)	17.58(90/512)	0.965
Live birth rates per transfer (%)	15.08(319/2116)	14.80(241/1628)	11.91(61/512)	0.183
Miscarriage rates (%)	16.27(62/381)	16.61(48/289)	32.22(29/90)#*	0.001
Cumulative live birth rates (per cycle)	28.50(603/2116)	24.94(406/1628)#	20.51(105/512)#*	< 0.001

#*P* < 0.05, vs. GnRH agonist

*P < 0.05, vs. GnRH antagonist

## Discussion

In assisted reproductive technology, the issue of poor ovarian response poses a considerable obstacle, particularly for women of advanced age [7, 20]. The implementation of appropriate controlled ovarian hyperstimulation protocols is crucial for improving pregnancy outcomes in this population. Our findings indicate that the modified GnRH agonist protocol is linked to a greater total dosage of gonadotropin administered and a longer duration of gonadotropin usage compared to the GnRH antagonist and Mild stimulation protocols. The higher total dosage of gonadotropin used in the GnRH agonist protocol increase the higher number of oocytes retrieved, higher number of mature oocytes, higher number of transferable embryos and good-quality embryos. However, our study revealed that there were no significant differences in pregnancy rates and live birth rates per transfer among the three protocols for patients aged 35 years and above with POR. Nevertheless, if patients necessitate additional frozen-thawed embryo transfers to enhance cumulative live birth rates, it becomes imperative to reassess the cost-effectiveness of the modified GnRH agonist protocol. This is due to the fact that the protocol group exhibited a significantly higher cumulative live birth rate compared to the GnRH antagonist and Mild stimulation groups.

Therefore, we propose implementing a modified GnRH agonist protocol for patients aged 35 and above with POR who require additional frozen-thawed embryo transfers in order to enhance cumulative live birth rates. It is crucial to exercise caution in order to prevent OHSS, as the agonist protocol exhibits a slightly higher incidence of OHSS compared to the other two protocols. Nevertheless, it is important to highlight that the occurrence of OHSS in each of the three categories is comparatively minimal (below 5%). This study aims to assess the efficacy of three distinct ovarian hyperstimulation protocols in patients undergoing ART treatment. The lack of significant variations in baseline characteristics among patients undergoing different ovarian stimulation protocols mitigates the potential confounding factors that could influence the comparison of these protocols’ effectiveness. Consequently, this ensures that any observed differences in outcomes are solely attributable to the variations in the protocols employed, rather than disparities in the patients’ baseline characteristics. Based on our findings, it is recommended that healthcare providers take into account the specific reaction of each patient to stimulation and select the ovarian hyperstimulation protocol that is best suited to attain the most favorable results.

Furthermore, our observations indicate that the utilization of the Mild stimulation protocol is linked to a heightened incidence of miscarriage compared to the GnRH agonist and GnRH antagonist protocols. The elevated rate of miscarriage observed within the Mild stimulation group is a matter of apprehension. Previous studies have reported a correlation between miscarriage and diverse factors, including maternal age and embryonic chromosomal abnormalities [21–23]. Nevertheless, the absence of age disparities among patients undergoing the various protocols in our study implies that the outcomes of these protocols may be comparable for patients of similar age. Similarly, body mass index, basal follicle-stimulating hormone (FSH), luteinizing hormone (LH), estradiol, and anti-Müllerian hormone (AMH) levels have been identified as significant predictors of ovarian reserve and spontaneous abortion [24, 25], however, further investigation is necessary to fully explore this potential relationship. Conversely, the administration of GnRH agonist protocol has been associated with a notably elevated cumulative live birth rate. A higher cumulative live birth rate is indicative of a greater success rate of the protocol in terms of achieving successful pregnancies that result in the birth of viable infants. This observation can be attributed to the capacity of GnRH agonist to inhibit LH secretion, thereby preventing premature ovulation and enhancing the quality of obtained oocytes [26]. In conclusion, our study posits that the Mild stimulation protocol may be linked to an elevated miscarriage rate, whereas the GnRH agonist protocol may be associated with a higher cumulative live birth rate. These findings offer valuable insights for further research and clinical practice.

Controlled ovarian hyperstimulation is a crucial component of ART, involving the administration of exogenous gonadotropins to induce the development of multiple follicles [14]. While COH has been shown to enhance the success rates of ART, the selection of an optimal protocol poses a significant challenge for clinicians [9, 27]. Historically, COH protocols have entailed the administration of high doses of follicle-stimulating hormone (FSH) and luteinizing hormone (LH) over an extended duration. However, this approach may lead to excessive oocyte production, ovarian hyperstimulation syndrome, and compromised oocyte quality. Furthermore, it has been observed that patients who solely undergo gonadotropin stimulation may face an increased likelihood of experiencing miscarriage and multiple pregnancies [28, 29]. A research study conducted by Koot et al. revealed that the inclusion of a GnRH antagonist in a GnRH-a protocol substantially enhanced the pregnancy rate among individuals undergoing ART, while also reducing the risks associated with OHSS and multiple pregnancies [30]. Nevertheless, despite the potential advantages offered by the protocol, the success rate of achieving pregnancy remains relatively low, and the most effective approach for older patients with POR remains uncertain [31, 32].

It is widely acknowledged that age significantly influences oocyte quality and embryo ploidy, both of which are crucial factors in determining the success of pregnancy [33]. Consequently, personalized protocols should be devised for these older individuals to address their unique requirements. Numerous studies have proposed the utilization of recombinant follicle-stimulating hormone (rFSH) as a potential strategy, as it has been demonstrated to provide more consistent stimulation and minimize the potential for exposure to contaminants compared to urinary-derived preparations. [34, 35]. Furthermore, the utilization of GnRH antagonist can effectively inhibit premature ovulation and enhance the recruitment of follicles. Adjuvant treatments, including the administration of growth hormone (GH), have been suggested as potential means to enhance ovarian response [36, 37]. GH has the potential to stimulate folliculogenesis and enhance oocyte quality, thereby increasing the likelihood of successful fertilization and implantation. Nevertheless, additional research is required to validate these observations [38].

In summary, the study findings indicate that there were no significant disparities in fresh-cycle implantation rates and live-birth rates when comparing the three ovarian stimulation regimens in older patients with POR. Nevertheless, it is worth noting that if patients aim to enhance cumulative live birth rates through additional frozen-thawed embryo transfers, the modified GnRH agonist protocol may be preferable. Additionally, it is important to highlight that the Mild stimulation regimen exhibited a slightly elevated miscarriage rate compared to the other two groups. However, it is crucial to acknowledge the existence of certain limitations within this study. Firstly, this study is characterized as a retrospective study, which may be susceptible to selection bias and incomplete data. Secondly, the study participants are exclusively sourced from a solitary medical institution, thereby potentially imposing regional and population limitations. Furthermore, the study lacks a comparison of the cost-effectiveness of various COH regimens, which further restricts the study’s comprehensiveness. Despite suggesting the potential benefits of the GnRH agonist regimen in elderly patients with POR, further prospective randomized controlled trials are imperative to validate its effectiveness and safety. These trials are essential in order to furnish more comprehensive evidence to inform clinical decision-making.

## Data Availability

The raw data supporting the conclusions of this article will be made available by the authors, without undue reservation, all data is available from the corresponding author on reasonable request. The important and representative information are available in the quotations and tables available in the manuscript.
